# May brood desertion be ruled by partner parenting capability in a polygamous songbird? An experimental study

**DOI:** 10.1002/ece3.11394

**Published:** 2024-05-13

**Authors:** Jia Zheng, Hui Wang, Jiayao Jiang, Maaike A. Versteegh, Zhuoya Zhou, Zhengwang Zhang, De Chen, Jan Komdeur

**Affiliations:** ^1^ Ministry of Education Key Laboratory for Biodiversity Sciences and Ecological Engineering, College of Life Sciences Beijing Normal University Beijing China; ^2^ Behavioral and Physiological Ecology, Groningen Institute for Evolutionary Life Sciences University of Groningen Groningen The Netherlands

**Keywords:** brooding duration, feeding frequency, parental removal, parenting capability, sexual conflict

## Abstract

Parents confront multiple aspects of offspring demands and need to coordinate different parental care tasks. Biparental care is considered to evolve under circumstances where one parent is not competent for all tasks and cannot efficiently raise offspring. However, this hypothesis is difficult to test, as uniparental and biparental care rarely coexist. Chinese penduline tits (*Remiz consobrinus*) provide such a system where both parental care types occur. Here, we experimentally investigated whether parents in biparental nests are less capable of caring than parents in uniparental nests. We monitored parenting efforts at (1) naturally uniparental and biparental nests and (2) biparental nests before and during the temporary removal of a parent. Given the relatively small sample sizes, we have employed various statistical analyses confirming the robustness of our results. We found that total feeding frequency and brooding duration were similar for natural uniparental and biparental nests. Feeding frequency, but not brooding duration, contributed significantly to nestling mass. In line with this, a temporary parental removal revealed that the remaining parents at biparental nests fully compensated for the partner's feeding absence but not for brooding duration. This reflects that the manipulated parents are confronted with a trade‐off between feeding and brooding and were selected to invest in the more influential one. However, such a trade‐off may not occur in parents of natural uniparental care nests. The different capabilities of a parent independently coordinating feeding and brooding tasks suggest that parents from biparental and uniparental nests were exposed to different resource conditions, thereby foraging efficiency may differ between care types.

## Graphical abstract

We explored differences between parents from uniparental care and biparental care nests through a temporary parental removal experiment with Chinese penduline tits. We observed no significant difference in the amount of care provided in natural conditions between the two nest types. However, when one parent was removed from biparental nests, the remaining parent increased food provisioning but could not match the lost brooding time. These results indicated that biparental care nests face a trade‐off between various parental duties, a challenge not observed in uniparental care nests.

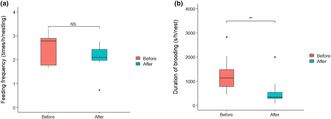

## 1 INTRODUCTION

In many species, the development of offspring largely relies on parental care, such as incubation, protection and food provisioning (Clutton‐Brock., 2019; Royle et al., 2012). The amount and quality of parental care patterns vary across and within species and are in part determined by the offspring's developing mode and number of caregivers. For instance, parental care is absent in most fish and reptiles since the offspring are able to move around freely after hatching and feed themselves. Conversely, in altricial birds, mammals and some frogs, parents have to provide intensive parental care due to the offspring's lack of self‐sufficiency (Balshine & Abate, 2021; Cockburn, 2006; Saraiva et al., 2012; Vági et al., 2019). In genetically monogamous species, males and females equally share genetic benefits (i.e. reproductive fitness) but bear the costs of parental investment on their own (Royle et al., 2012). Under these circumstances, both parents should induce the other to invest more than themselves, regardless of uniparental or biparental (Székely, 2014). The number of caregivers providing parental care reflects the different manners of engagement in mandatory cooperative work (i.e. breeding) in animals with various biological backgrounds.

Theoretical studies have shown that uniparental care is more likely to evolve as a stable strategy when the abandoned partner is capable of successfully raising the offspring alone (Klug & Bonsall, 2010; Webb et al., 2002). Accordingly, deserters in some species may evaluate whether their partner is competent to handle the remaining feeding tasks alone when deciding to desert (Kupán et al., 2021; Roulin, 2002). This may explain why offspring desertion usually takes place in the feeding period when offspring can gain sufficient care from single parents (Béziers & Roulin, 2016; Kupán et al., 2021). In contrast, declined reproductive success was also found after one parent had deserted the dependent offspring (Cruz‐López et al., 2017; Griggio & Pilastro, 2007; Székely et al., 1999). To date, it is unknown whether an individual's deserting decision is ruled by the parenting capability of its partner, due to the lack of experimental studies in wild species (for observational studies, see Kupán et al., 2021; McDonald et al., 2023).

Furthermore, due to the large reliance of offspring fitness (i.e. development and survival) on parental attendance, parents must simultaneously take charge of multiple care tasks, such as nest protection, nestling feeding and brood temperature regulation, during the parenting process (Royle et al., 2012; Vági et al., 2019). Therefore, parents need to coordinate various parental care tasks during offspring rearing stages (Eldegard & Sonerud, 2009; Mock, 2022). When parents are confronted with a trade‐off in offspring rearing, they may especially invest in those parental care tasks that are most important for nestling fitness (Wischhoff et al., 2018). Food provisioning and nestling brooding in avian species are two crucial parental tasks, where parents need to manage their efforts either inside or outside the nest. How well single parents in uniparental and biparental care nests cope with these spatially conflicting tasks in terms of time arrangement could reflect their parenting capability (Heaney & Monaghan, 1996).

Uniparental care (either male or female‐only care) often coexists with biparental care in the few species showing offspring desertion (Gonzalez‐Voyer et al., 2022; Pilastro et al., 2001; Roulin, 2002; Székely et al., 1996; Zheng et al., 2018). This type of system provides a great opportunity to compare the parental ability of parents in naturally uniparental and biparental care nests. More specifically, observing the parental effort from the remaining parents after the removal of one parent will elucidate whether parents in biparental care nests are less capable in caring than parents in uniparental care nests, thereby impeding the occurrence of desertion in biparental nests. Exploring whether the remaining single parent can (over) match the care provided by both parents has been commonly conducted in biparental species (Harrison et al., 2009; Lou et al., 2021; Ringler et al., 2015; Royle et al., 2002). Actually, by temporarily removing the partner, the remaining parent faces a new situation where it needs to not only adjust its parental effort but also reallocate its effort to those parental care tasks that are most important for nestling fitness. By monitoring the change in parental effort of the remaining parent before and after removing a parent, the capability of a parent to independently raise the brood will be revealed.

The aim of our study was to test whether decision rules for parental desertion may involve the parental ability of the remaining parent. We used the Chinese penduline tit (*Remiz consobrinus*) as our model species because biparental (60% of nests), female‐only (30% of nests) and male‐only care (10%) occurs in the same population (Zheng, 2022; Zhou, 2022). Chinese penduline tits desert the clutch at the early stage of incubation, which means that the parental care type is fixed for a nest during the entire nestling feeding stage. Therefore, this species is ideally suited for carrying out temporal parental removal experiments. Our study tries to answer the following questions: (1) Do feeding frequency and brooding duration differ between naturally biparental care and uniparental care nests, and if so, how? (2) How is each care task associated with nestling fitness? (3) Can the remaining parent fully compensate for the effort of the partner that has been removed? (4) Female‐only care is generally more prevalent in Chinese penduline tits than male‐only care nests (Zheng et al., 2018). Do the two sexes feed and brood differently in biparental care nests, and do they compensate differently in response to the removal of their partner?

## 2 METHODS

### 2.1 Study site

Our study was conducted in Xianghai National Reserve, Jilin Province, China (44°55′–45°09′ N, 122°05′–122°31′ E), an inland area. The migratory Chinese penduline tits arrive at the field site and breed from the end of April until August (Tong et al., 1985; Zhou, 2022). The main tree species are large‐fruited elm (*Ulmus macrocarpa*), Siberian elm (*Ulmus pumila*), willow (*Periploca sepium*) and Chinese white poplar (*Populus tomentosa*), which are distributed across the plain meadows (Zhou, 2022). Chinese penduline tits build their nests on the outer branches of these trees.

### 2.2 Basic breeding monitoring and parental provisioning

We conducted fieldwork from May 1st to August 10th in the years 2019, 2020 and 2021. We searched the study area for newly built nests daily by inspecting tree branches and tracking the songs of male penduline tits. The nest stage (see Zheng et al., 2018, Figure 1a–f) was recorded once a nest was found, and the development of that nest was checked every 2 days. When a nest was developed to stage D (when nest was shaped as a basket, Tong et al., 1985; Zheng et al., 2018), we began checking for the presence of eggs every day by carefully hand‐touching the inside of the nest. In this way, we were able to feel the egg shape underneath the burying material without damaging the structure of the burying layer. Since the clutch size ranges from six to nine eggs and penduline tits lay one egg every morning, we thereby confirmed the clutch size 10 days after the day of the first laid egg. Hatching was checked from the 11th day of incubation onwards. If we found hatchlings in the morning, the day was recorded as the first day of hatching, and nestling age was recorded as Day 0. In Chinese penduline tits, parental care patterns of nests are often determined by parents at the onset of incubation (Zheng et al., 2018). We filmed the nest with a SONY CX680 video camera of the nestlings at 4 days old for 2.5 h to confirm the parental care pattern for each nest (biparental, male‐only or female‐only care). To compare the parental provisioning between biparental care nests and uniparental care nests, we filmed nestling feeding frequency with video cameras also when nestlings were aged 8, 12 and 16 days for the same length of time. Cameras were fixed on a tripod at 40 centimetres high and 3–5 m away from the tree trunk. The camera settings were wrapped with a camouflage cloth that was similar to the environmental background, with only the lens exposed to prevent disturbance of bird activities. We adjusted the focal length until the nest was entirely contained in the close‐up shooting frame. In this way, we were able to clearly record the feeding events and differentiate between the parents (females have brown eye masks and heads, males have black eye masks and grey heads).

**FIGURE 1:**
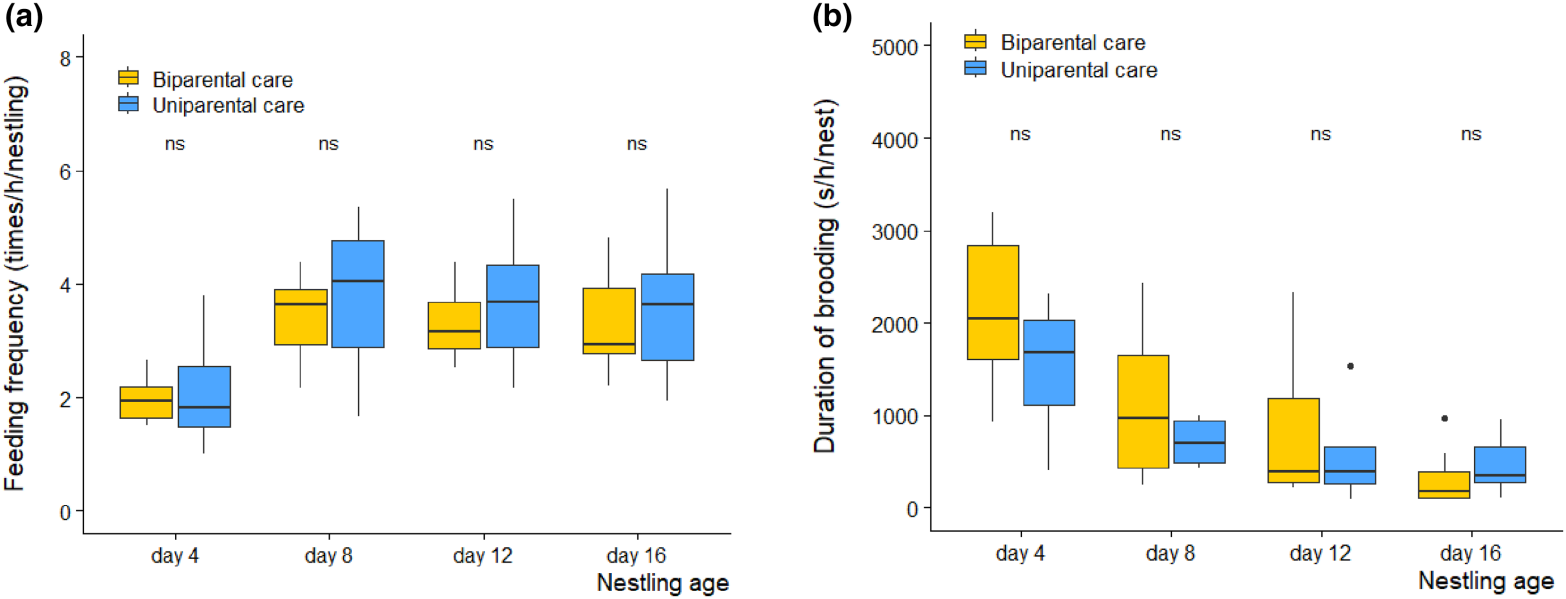
Mean parental (a) feeding frequencies and (b) brooding duration of Chinese penduline tits for biparental care (male + female) and uniparental care nests over nestling age (20 nests, 10 nests biparental care, 10 nests uniparental care; 80 observations). Thick horizontal lines indicate the medians, whereas the bottom and top of the boxes are the 25th and 75th quartiles, respectively. Numbers above each box indicate sample sizes, and dots show the outliers; ns, not significant. There was no significant difference in feeding frequency between uniparental care and biparental care nests over nestling growth (for statistics, see Table 1).

The recordings were conducted in the morning when birds were active. If the planned filming day was rainy, we postponed filming for 1 day. We selected nests at a maximum height of 6 m on the trees (84% of the nests) because we could not reach higher nests. The number of nestlings in the nests on the day of filming was also checked once the filming had finished. We selected only biparental nests and female‐only care nests since the number of suitable male‐only care nests was low (10% of all active nests). We counted the number of hatchlings 3 days after the first nestling hatched (Zheng et al., 2021). We measured the body mass of nestlings aged 5, 9, 13 and 17 days. The number of fledglings was confirmed on nestlings aged 17 days (nestlings fledge around age 21 days, Zheng et al., 2018). Fledging success was calculated by the number of fledglings divided by the number of hatchlings.

### 2.3 Parental removal experiment

We carried out the temporary parental removal experiment (temporarily removed seven females and four males from the 11 experimental nests) using biparental nests on 1 day between nestlings when aged 6–9 days. Removal was only conducted in the morning (i.e. 6–11 am) of a sunny day to minimise possible interference of the experiment on nestling survival. First, we filmed the nests for 2.5 h to determine the nestling feeding frequency under normal conditions. After filming, we trapped one parent with a mist net (6 m × 3 m) and left the caught bird in a cage for the duration of the experiment.

The birdcage was situated at a location far away (>200 m) from the nest so that the other parent (focal parent) was unable to see and hear the removed parent to communicate. We covered the birdcage with a dark‐green cloth to decrease disturbance of the removed parent. We continued to film the focal nest for no less than 2.5 h after bird catching to monitor the feeding frequency of the focal parent after removing the partner. All focal birds fed and brooded nestlings for the first time within 30 min after partner removal (20.2 ± 7.8 min on average ± SD; *n* = 10 nests). The removed partner was released after 2.5 h of nest filming. We never witnessed two birds trapped simultaneously in the net. None of the birds deserted the nest during or after the entire experimental procedure. The short period of parental removal did not influence the fledging success of experimental nests compared with unmanipulated biparental nests (*t* = 1.62; *p* = .12; *n* = 11 experimental biparental nests, *n* = 12 unmanipulated biparental nests). The recruitment rate of Chinese penduline tits is very low (Zheng et al., 2021), we never removed the same bird twice for this experiment over the years. No significant variation in feeding frequency (*t* = 0.63, *p* = .52, *n* = 19 nests) and brooding duration (*t* = 0.17, *p* = .87, *n* = 19 nests) was found regarding the start time of filming in the morning.

### 2.4 Data analyses

All feeding videos were analysed with Behavioural Observation Research Interactive Software (BORIS v.7.7.5; Friard & Gamba, 2016). We disregarded the first 0.5 h of each feeding video to exclude potential disturbance of parental feeding as a consequence of researchers setting up the camera. We recorded the times per hour of male and female feeding nestlings and the duration per hour of each feeding time within the 2‐h video. Statistical analyses were performed using R version 4.1.1 (R Core Team, 2021), and the null hypotheses were rejected at *p* < .05. Mean ± SD are provided in the results. We used the ‘dredge’ function (‘MuMln’ package) to select the optimal models that exhibited the smallest AIC (ΔAICc < 2) among the combinations of variables for our analyses (Appendix S1).

#### 2.4.1 Comparisons of parental provisioning

One feeding time was recorded when we observed a parent directly flying into the nests. For each nest, we calculated the feeding frequency (times per hour per chick) using the equation below:

$$\text{Feeding frequency}=\frac{\text{Feeding times observed}}{\text{video length}\;\left(h\right)\times \text{number of chicks}\;}$$

Brooding duration was recorded as the time period from the moment a parent returned to feed in the nests until it left. We calculated the brooding duration (seconds per hour) for each nest with the equation below:

$$\text{Brooding duration}=\frac{\text{Total feeding duration observed}\;\left(s\right)}{\text{video length}\;\left(h\right)}$$

We built a linear mixed model (LMM) for analysing the difference in feeding frequency and Gamma generalised linear mixed models (GLMM) for analysing brooding duration between uniparental care nests and biparental care nests over nestling ages. Feeding frequency and brooding duration (which was log transformed) were respectively the response variables, and parental care type, nestling age and their interaction were the predictors. The number of nestlings was used as an additional predictor in the model for brooding duration. The year and the number of nestlings were not included as predictors in the final model, as they both have no effect on the response variables during the process of sieving for the optimal models.

To investigate whether male and female feeding frequencies differed in biparental care nests, we built up (1) a linear mixed models (LMM) with feeding frequency as response variables, and (2) a Gamma generalised linear mixed model (GLMM) with brooding duration (which was log transformed) as response variable; sex, nestling age as the predictors, and nest ID was as the random factor in these two models. Year, the number of nestlings was not included as predictor in the final model due to the absence of effects on the response variables. The interactions between nestling age and care types (*p* = .75) and between nestling age and parental sex (*p* = .32) were also excluded from the model because of the lack of significant effects. To explore whether feeding frequency and brooding duration are related to nestling development and survival, we created: (1) an LMM with nestling body mass as the response variable, and feeding frequency, brooding duration, and nestling age as the predictors, with nest ID as the random factor. There is no multicollinearity between brooding duration and feeding frequency (VIF < 1.5). (2) A GLM with binomial errors using the number of hatchlings that survived and those that did not as the response variable with feeding frequency and brooding duration as the predictors. Most offspring that survive until Day 16 successfully fledge (Zheng et al., 2018, 2021). Therefore, fledging success was calculated as the number of surviving offspring on Day 16 divided by the number of hatchlings of the brood. The effect of care type was excluded from the two models (p1 = 0.44 and p2 = 0.79) because of the lack of influence on the above‐mentioned response variables.

#### 2.4.2 Parental provisioning before and after the parental removal experiment

To compare the differences in parental efforts before and after removing one parent, we built up an LMM for analysing feeding frequency and a Gamma GLMM for analysing brooding duration. (1) Total feeding frequency and (2) total brooding duration at a nest before and after removing one parent were the response variables respectively for the two models. Brooding duration was log transformed. Manipulation stage (before or after removal), non‐removed sex were the predictors, and nest ID was the random factor. The interaction between the manipulation stage and non‐removed sex had no effect on the response variables (p1 = 0.77, p2 = 0.86) and therefore was not included in the model.

To exclude the potential bias that may occur because we more often caught the bird that was more active in feeding and thus had a higher chance of being caught close to the nest, we created a generalised linear model using logistic regression with the sex of the caught parent as the response variable and the ratio of female feeding/total feeding of each nest before removal as the predictor. There was no association between feeding frequency and the probability of being caught (*n* = 10 nests; *p* = .57), and we did not consider the bias in bird catching for the rest of our analyses.

### 2.5 Testing for statistical robustness

Regarding the relatively small sample size of nests monitored in our study (*n* = 20 for natural nests, *n* = 11 for experimental nests), we first conducted a power analysis with the ‘pwr’ package to analyse the probability of true statistical effects that we have drawn from all the aforementioned statistical models. Later, we conducted Bayesian analyses by using ‘brm’ package to build linear multilevel models with the same response variables, predictors and random factors as in the aforementioned statistical models. We compared the statistical results from these two types of models.

### 2.6 Ethical note

#### 2.6.1 Informed consent

Informed consent was obtained from all individual participants included in the study.

## 3 RESULTS

### 3.1 Comparisons between care types: Feeding and brooding behaviour

**FIGURE 2:**
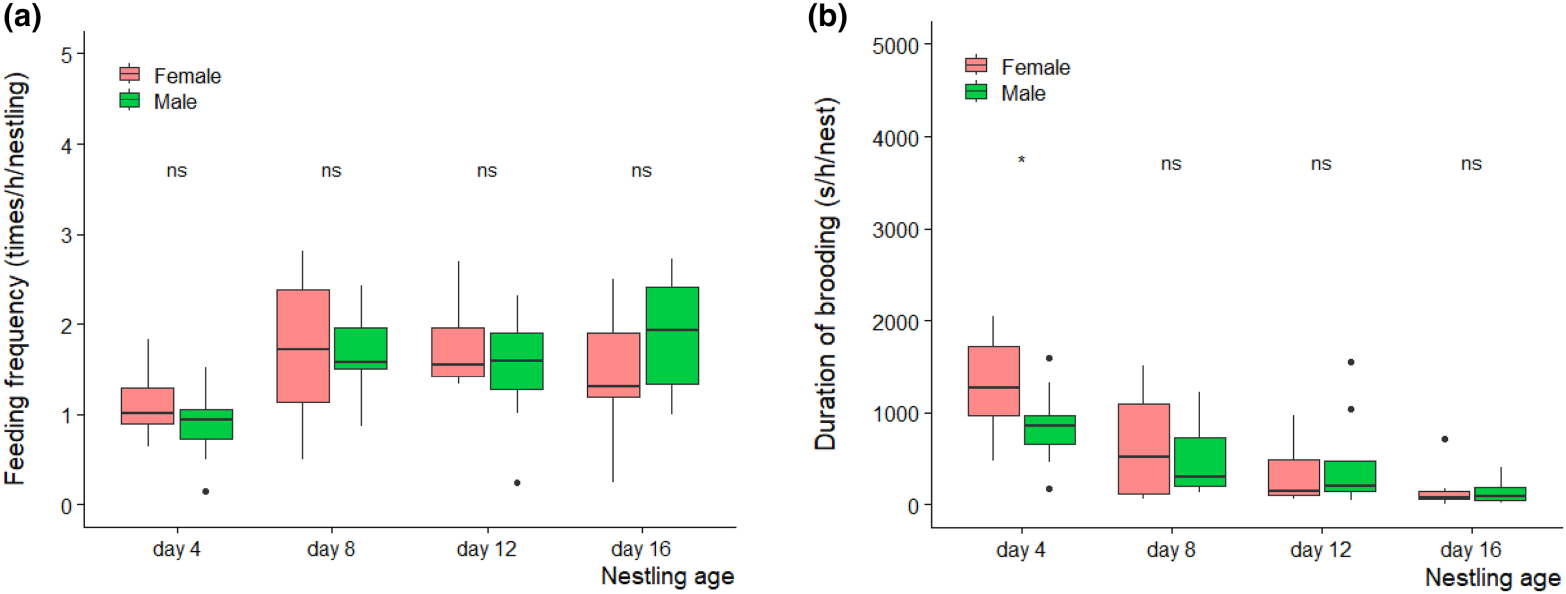
Mean (a) feeding frequency and (b) brooding duration of female and male Chinese penduline tits of biparental care nests over nestling age (*n* = 10 nests; 40 observations). Thick horizontal lines indicate the medians, whereas the bottom and top of boxes are the 25th and 75th quartiles, respectively. Numbers above each box indicate sample sizes, and the dots show the outliers; ns, not significant. For statistics, see Table 2.

The feeding frequencies increased over nestling ages but were not different between the uniparental and biparental care nests (Table 1, Figure 1a). Year had a significant effect on feeding frequency (Table 1). In biparental care nests, we did not find difference in feeding frequency between male and female parents, and the frequencies significantly increased over nestling age (Table 2, Figure 2a). The brooding duration declined over nestling ages, but were not different between uniparental and biparental care nests over nestling ages (Table 3, Figure 1b). Brooding duration was not associated with the number of nestlings in the nests (Table 3). In biparental care nests, male and female parents spent an equal amount of time brooding for a given nestling age (except 4 days old, Figure 2b), but their brooding duration decreased with nestling age (Table 4, Figure 2b).

**TABLE 1:** Feeding frequency in relation to parental care type (uni‐ versus biparental care) and nestling age in Chinese penduline tits. GLMM was used with nest ID as the random factor (*n* = 20 nests, 80 observations).

Response variable	Predictors	Estimate	SE	*t* Value	Pr (>|*t*|)	*R* ^2^
Feeding frequency	Intercept	2.50	0.34	7.42	<.001	.43
	Care type	0.388	0.26	1.51	.15	
	Nestling age	0.11	0.02	4.61	**<.001**	
	Year	−0.93	0.29	−3.25	**<.01**	

*Note*: Bold values indicate p < .05 considered as statistically significant.

**TABLE 2:** Statistics of feeding frequencies of male and female Chinese penduline tits in biparental nests. GLMM was used with nest ID as the random factor (*n* = 10 nests, 40 observations).

Response variable	Predictors	Estimate	SE	*t* Value	Pr (>|*t*|)	*R* ^2^
Feeding frequency	Intercept	0.99	0.19	5.31	<.001	.20
	Sex	−0.05	0.14	−0.35	.73	
	Nestling age	0.05	0.02	3.22	**<.01**	

*Note*: Bold values indicate p < 0.05 considered as statistically significant.

**TABLE 3:** Brooding in relation to parental care type (uni‐ versus biparental care) and nestling age in Chinese penduline tits. GLMM was used with nest ID as the random factor (*n* = 20 nests, 80 observations).

Response variable	Predictors	Estimate	SE	*t* Value	Pr (>|*t*|)	*R* ^2^
Log (brooding duration)	Intercept	0.12	0.01	19.14	<.001	.60
	Care type	−0.004	0.006	−0.69	.49	
	Nestling age	0.003	0.001	7.90	**<.001**	

*Note*: Bold values indicate p < 0.05 considered as statistically significant.

**TABLE 4:** Brooding duration of male and female Chinese penduline tits at biparental care nests. GLMM was used with nest ID as the random factor (*n* = 10 nests, 40 observations).

Response variable	Predictors	Estimate	SE	*t* Value	Pr (>|*t*|)	*R* ^2^
Log (brooding duration)	Intercept	0.12	0.01	11.71	<.001	.53
	Sex	0.002	0.007	0.26	.79	
	Nestling age	0.005	0.001	6.46	**<.001**	

*Note*: Bold values indicate p < 0.05 considered as statistically significant.

### 3.2 Implications of feeding and brooding behaviour on nestling fitness

**FIGURE 3:**
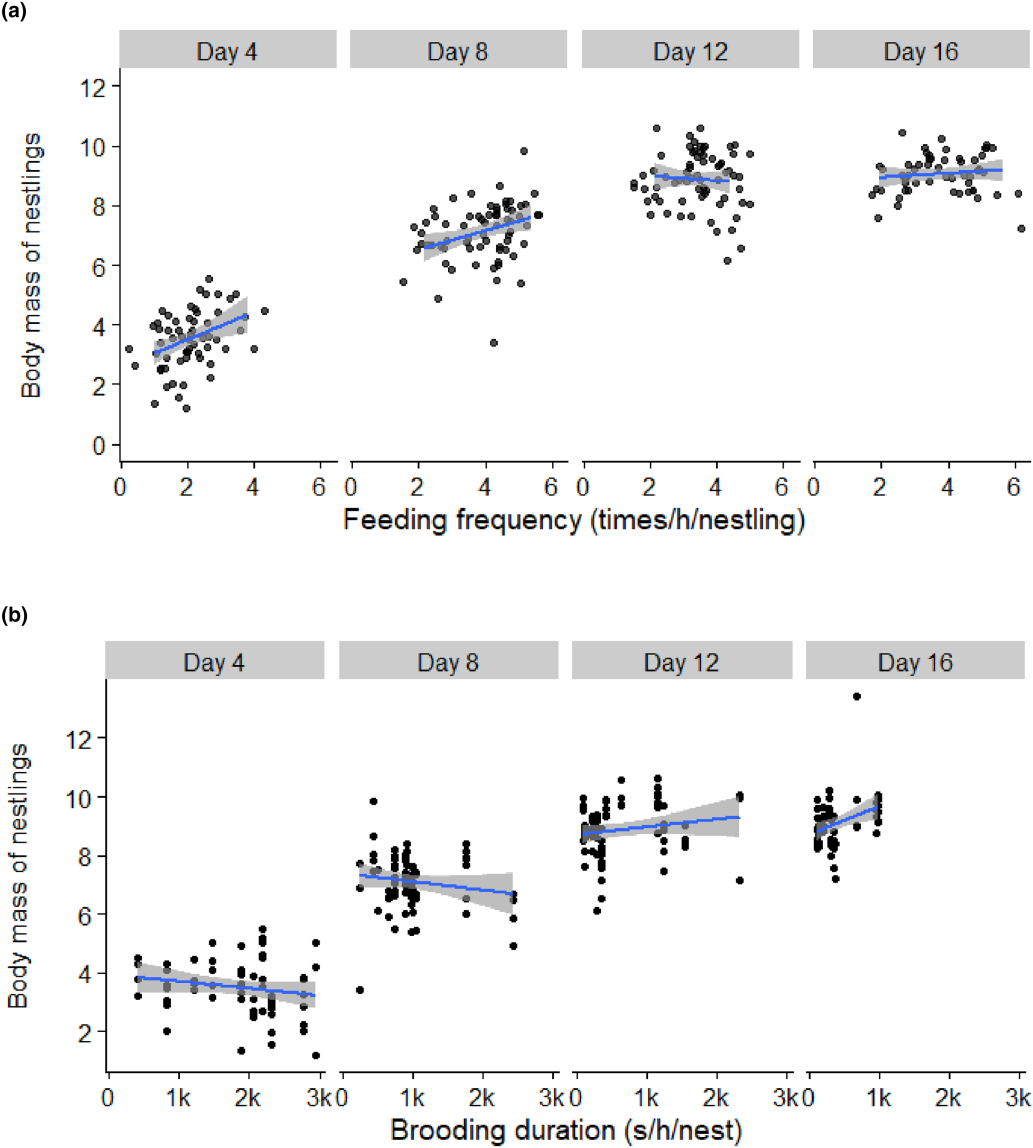
The relations of (a) feeding frequency and (b) brooding duration to nestling body mass in Chinese penduline tits (*n* = 14 nests). The black dots indicate the body mass of one nestling under a certain feeding frequency at a certain day age. The blue solid lines describe the linear trend of the relationship between variables on the x‐ and y‐axes. The grey shades indicate the 95% confidence intervals. For statistics, see Table 5.

The body mass of nestlings was positively associated with feeding frequencies across all nestling stages but not with brooding duration conducted by parents regardless of the care type of nests (Table 5a). The influences of feeding frequency and brooding duration on nestlings' body mass were variable over nestling ages, and importantly, their influences on body mass were negatively related (Table 5a, Figure 3). The fledgling success of nestlings was not associated with either feeding frequency or brooding duration and was independent of the care type of nests (Table 5b).

**TABLE 5:** Association of feeding frequency and brooding duration with nestling development (a. body mass; b. fledging success) in natural nests. GLMM was used with nest ID as the random factor (*n* = 14 nests, 68 nestlings).

Response variable	Predictors	Estimate	SE	*t* Value	Pr (>|*t*|)	*R* ^2^
(a) Body mass of nestling	Intercept	3.26	0.70	4.67	<.001	.84
	Feeding frequency	0.23	0.08	3.01	**<.01**	
	Log (Brooding duration)	−0.02	0.09	−0.24	.81	
	Nestling age	1.43	0.09	15.38	**<.01**	
(b) Fledgling success	Intercept	3.97	1.32	2.80	<.01	.25
	Feeding frequency	0.01	0.83	0.02	.99	
	Log (Brooding duration)	0.02	0.18	0.11	.91	

*Note*: Bold values indicate p < 0.05 considered as statistically significant.

### 3.3 Influence of parental removal on provisioning and brooding by the remaining parent

**FIGURE 4:**
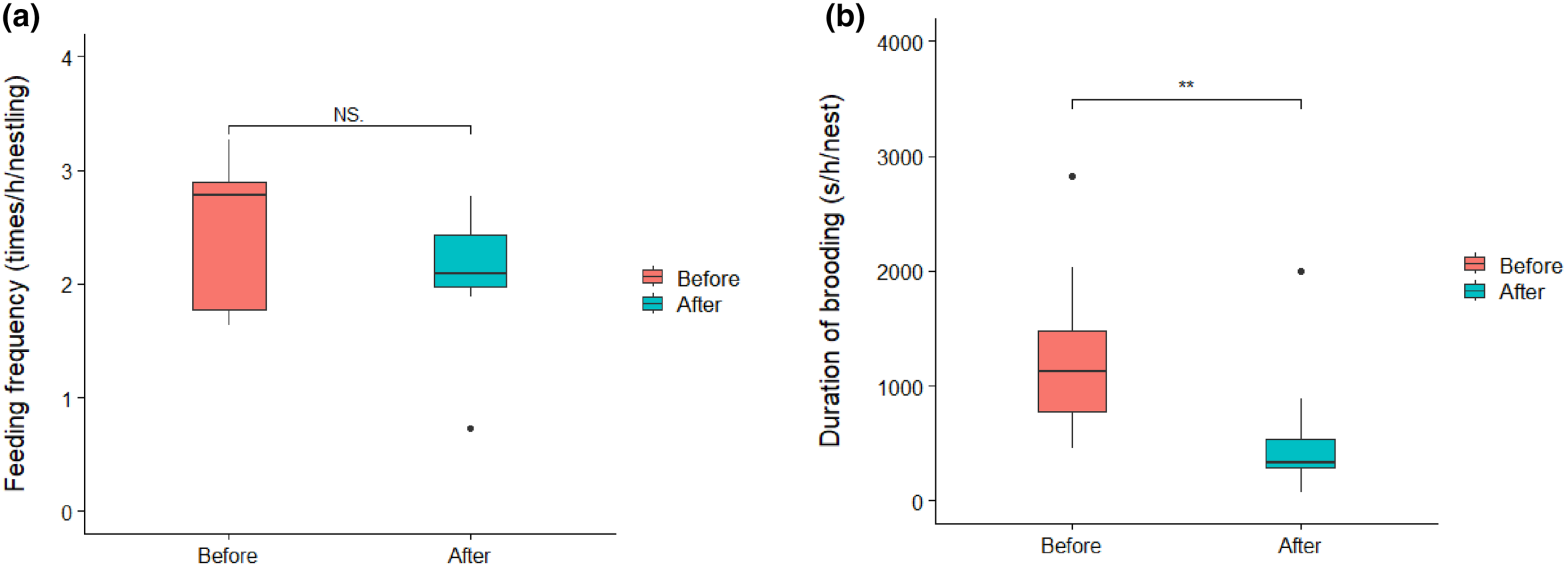
Mean (a) feeding frequencies and (b) brooding duration by the breeding pair of biparental care nests before and by the remaining parent after parental removal in Chinese penduline tits (*n* = 11 nests; 22 observations). Thick lines indicate the medians, whereas the bottom and top of boxes are the 25th and 75th quartiles, respectively. Numbers above each box indicate the sample sizes, and the dots show the outliers; ns, not significant. *p < .05 and **p < .01. For statistics, see Table 6.

Parental removal experiments could test the presence of the trade‐off between feeding and brooding given that feeding frequency is more important for nestling fitness than brood duration. We found that the remaining parents could fully compensate for the absence of feeding of its removed partner (mean ± SD: 0.92 ± 0.36 times the original feeding rate from both parents, Table 6a, Figure 4a). After removing a parent from biparental care nests, the remaining parent significantly increased its nestling feeding rate (mean ± SD: 1.77 ± 0.81 times the original feeding rate). The compensation of feeding frequency did not differ between a remaining male or female (Table 6a).

**TABLE 6:** Comparison of the compensation for feeding frequency and brooding duration during the parental removal experiment in Chinese penduline tits with the total feeding rate and brooding duration of both parents before the experiment. GLMM was used with nest ID as the random factor (*n* = 11 nests, 22 observations).

	Estimate	SE	*t* Value	Pr (>|*t*|)	*R* ^2^
(a) Provisioning frequency					
Intercept	2.43	0.20	11.98	<.001	.10
Manipulation stage	−0.35	0.25	−1.39	.18	
Sex	0.09	0.26	0.33	.74	
(b) Log (brood duration)					
Intercept	0.15	0.01	22.09	<.001	.72
Manipulation stage	0.03	0.01	4.78	**<.001**	
Sex	−0.02	0.01	−1.49	.137	

*Note*: Bold values indicate p < 0.05 considered as statistically significant.

The overall duration of brooding significantly decreased after removing one of the parents (mean ± SD: 0.42 ± 0.31 times the original brooding duration from both parents, Table 6b, Figure 4b). The remaining parents did not compensate for the brooding duration after the partner was removed (mean ± SD: 1.12 ± 0.63 times the original brooding duration, *n* = 11 nests). There was no difference in brooding compensation between sexes (Table 6b).

The extent of compensation on feeding frequency and brooding duration response varied between individuals (extent of compensation: feeding, *t* = 8.70, *p* < .001; brooding, *t* = 5.90, *p* < .001). After removing one of the parents, 4/11 of the focal parents overcompensated in feeding frequency, where the compensated frequency was higher than the natural frequency from both parents; 5/11 of the focal parents partially compensated, where they increased feeding frequency but less than the naturally biparental frequency, and the other 2/11 kept the feeding rate consistent before and after removal (Appendix S1a). Regarding the brooding duration, only 2/11 of the remaining parents fully compensated for brooding, 4/11 of the parents almost did not change the time of brooding, and the remaining 5/11 of the parents even decreased their brooding duration (Appendix S1b).

### 3.4 Analyses for model robustness

We conducted power analysis for the statistical models showing significant effects. The probability of true effect(s) (1) for the model that compared feeding frequency (Table 1) and brood duration (Table 3) between naturally uniparental and biparental care nests were 83% and 99.8%, respectively; (2) for the model that analysed the associations of feeding frequency and brooding duration with nestling body mass was 99.9% (Table 5a); (3) for the model that compared brooding duration before and after partner removal was 85% (Table 6b); (4) for the model that compared feeding frequency and brood duration between the two sexes in naturally biparental care nest were 20% and 67% (Table 2 and Table 4). The results of Bayesian analysis showed similar estimation to the aforementioned (generalised) linear mixed models (shown in Appendix S3).

## 4 DISCUSSION

Our study revealed that in Chinese penduline tits, total parental provisioning and brooding for nestlings were similar for nests with different care types – biparental care versus uniparental care. Feeding frequency significantly contributed to the body mass of offspring, but brooding duration did not; neither influenced fledging success. Parental removal experiments executed in biparental care nests of Chinese penduline tits indicated that the remaining parent fully compensated for the feeding frequency in the absence of its partner but did not for the brooding duration. Parents may be confronted with a trade‐off in allocating parental efforts while dealing with the partner's absence, and they allocated more effort to the more influential one for offspring fitness. Sex‐biased feeding and brooding effort were not found in naturally biparental nests or in compensation for the partner's absence. These results suggest that the evolution of biparental care in Chinese penduline tits may not be driven by the low feeding capability of the partner but related to the ability of the partner to efficiently arrange different parental tasks.

Although the sample size of this research was relatively small, it still constituted a lot of research time, as we did multiple observations per nest during the nestling development stages. Therefore, increasing the sample size will not be easy. Moreover, the statistical power analysis we have done shows in general high power of effects of the models. The results of Bayesian analysis are also in line with our statistical results. This indicated that the data on this limited number of nests is solid for drawing the main statistical conclusions of this study. We expect these conclusions could provide insights into understanding the formation of various parental care patterns in penduline tits.

### 4.1 Different responses to parental removal in species with and without natural uniparental care

Uniparental care does not commonly evolve in birds, especially in passerine species, where nestlings demand close attendance from both parents (Cockburn, 2006). In most species where both care strategies occur, uniparental care is more likely an optional parenting strategy. Its occurrence has been found to be influenced by resource abundance, skewed adult sex ratio or energy reservation for moulting and migration in the late season (Eberhart‐Phillips et al., 2018; Eldegard & Sonerud, 2009; Wojczulanis‐Jakubas & Jakubas, 2012). However, studies have scarcely explored whether parents invest differently under uni‐ and biparental care conditions (Porkert & Špinka, 2004; Wiebe, 2005). Our results revealed that the single parent in uniparental care nests invests as efficiently as both parents in biparental care nests regardless of feeding or brooding. This indicates that the overall parental effort did not differ in the two types of nests. This result was consistent with the findings in common redstart (*Phoenicurus phoenicurus*; Porkert & Špinka, 2004), magnificent frigatebird (*Fregata magnificens*; Osorno & Székely, 2004) and Tengmalm's owl (*Aegolius funereus*; Eldegard & Sonerud, 2009). Those parents who were naturally deserted by the partner were able to fully compensate for the feeding loss to the same standards as biparental nests.

Our parental removal experiment found that in biparental care nests of Chinese penduline tits, one parent could completely compensate for the absence of food provisioning. This indicates that in a natural scenario with biparental care nests, the two parents resolved sexual conflict by withholding their provisioning effort from the maximum under the condition of guaranteeing sufficient care to the offspring (Hardling & Kaitala, 2005; Parker, 2006). Moreover, consistent with some theoretical predictions (Fromhage & Jennions, 2016; Houston et al., 2005), male and female penduline tits resolved the conflict by reaching conditional cooperation through evenly sharing the feeding and brooding task.

Conversely, in zebra finch (*Taeniopygia guttata*), a species that provides biparental care only, a study showed that after experimental removal of one parent during provisioning from some nests, broods reared by one parent were fed significantly less frequently than those raised by two parents (Royle et al., 2006). A meta‐analysis on parental response to experimental reductions in partner provisioning efforts found that the mean response for most biparental care species was not full compensation (Harrison et al., 2009). The conclusions drawn above are based on species where biparental care is the norm and in which a parent was experimentally removed to force a uniparental approach. This finding, together with our results, implies that uniparental care probably evolved as a stable pattern only when the efficiency of raising offspring by a single parent was relatively high.

### 4.2 Parents from biparental care nests confront a trade‐off during singly feeding

Our study investigated the parenting capability of parents from natural uni‐ and biparental nests in Chinese penduline tits by removing one parent from biparental care nests. The full compensation of nestling feeding during partner removal indicated that the parents in biparental nests may not necessarily have a lower ability in food provisioning than parents from uniparental nests. However, the remaining parent did not compensate for the reduced brooding duration. This agrees with the claim that comparable provisioning rates could result in less time spent on brooding nestlings (in common redstart; Porkert & Špinka, 2004).

Our removal experiment may force the remaining parents into a trade‐off such that compensation for feeding comes at the cost of brooding. This unequal compensation of the two parental tasks could be explained by the significant contribution of feeding frequency, but not brooding duration, to nestling body mass that we found in natural nests. Adjusting parental effort while facing a parenting trade‐off has been shown, for example, in common terns (*Sterna hirundo*), where parents increasing incubation effort for better brood hatching success had reduced parental performance in the later brood‐rearing phase (Heaney & Monaghan, 1996). In burying beetles (*Nicrophorus orbicollis*), parents allocate more time to care behaviours that offspring receive individually (such as feeding) but not simultaneously (such as brooding, Rauter & Moore, 2004). These findings, which are in line with ours, manifest that different parental care tasks may contribute different weights to offspring development, and parents tend to make the (temporal) sacrifice that deprives the least necessary of offspring fitness.

However, we found this parenting trade‐off may no longer hold in natural uniparental care nests, where single parents could easily feed and brood at the same intensity as the pair in biparental care nests. The inconsistency of parental care by single parents between natural‐ and manipulated‐uniparental care nests indicates that some fundamental differences in uniparental and biparental nests exist. For instance, (1) parents in uniparental care nests may occupy better territories, with more abundant food than in biparental care nests, so that single parents spend less time foraging and are therefore able to spend a sufficient amount of time keeping nestlings warm; (2) parents in biparental care nests are less efficient in foraging than parents in uniparental care nests. In penduline tits, uniparental care nests often appear early in the breeding season, whereas biparental care nests emerge later (Zheng, 2022). Possibilities can be that as food resources decline over the season (which could be indicated by a seasonally decreased clutch size; Zheng et al., 2021), the late parents have to cooperate to successfully raise nestlings; additionally, an intense breeding peak of penduline tits in the early season might allow individuals to socially share resource information and thereby forage more efficiently (Brandl et al., 2019; Evans et al., 2009). This may also make parents from the early nests (largely uniparental care nests) more capable of feeding on the brood alone; (3) the body condition of parents from uniparental and biparental care nests could also influence their breeding performance. For instance, parents with good body condition may be more capable of raising offspring alone (Bleeker et al., 2005; Seress et al., 2020). Further examination of physiologic parameters (e.g. immune competence, hormone levels) could assist in enhancing our understanding of this subject.

One may argue that the genetic relationship between parents and offspring may also contribute to differences in investment between uniparental and biparental care nests. Specifically, nests cared for solely by females may exhibit higher levels of extrapair paternity (EPY). Consequently, these females might be more inclined to increase their brooding and feeding efforts compared to females of biparental nests. This increased investment aims to boost their fitness by increasing the genetic diversity of their offspring (Ball et al., 2017; Sakamoto et al., 2023). However, this explanation does not hold true in Chinese penduline tits, as the percentages of nests with at least one EPY were similar between uniparental care and biparental care nests in our population (EPC occurring in 23.4% of the nests, uniparental care vs. biparental care: *χ* ^2^ = 2.95, df = 2, *p* = .23, *n* = 47 nests) (van der Velde M., Wang H, unpublished data). The consequences of the shorter brooding duration in experimentally created uniparental nests require more investigation, such as by monitoring the physiological conditions of offspring during experiments. In addition, despite the full compensation for food provisioning during the 2.5 h of parental removal, we could not conclude whether the remaining parents are fully competent for parental care alone or if the response changes from full to partial compensation. Long‐term removal should be considered, preferably over the entire nestling period. Based on the current results, we propose that studies should note that feeding ability, although probably the easiest measure to estimate, may not be the only parenting behaviour that matters if investigating the compensation for the partner's care. The restrictions for parents to allocate time and energy to different parenting tasks need more investigation in further studies. Biparental care may be necessary for providing a good environment for nestling development even if one of the parents is in principle able to provision the brood sufficiently in biparental care nests (Matysioková & Remeš, 2014; Rossmanith et al., 2009).

### 4.3 No sex difference in response to partner removal

Our study showed that the change in food provisioning of the remaining parent before and after removing the partner was similar for male and female parents. However, sexually different responses to the absence of partners were reported in other species. Male rock sparrows allocate more care to offspring and feed at a higher frequency after being widowed, whereas females do not adjust their feeding frequency when confronted with the absence of the male partner (Cantarero et al., 2019). Male and female northern flickers (*Colaptes auratus*) increase the amount of food provisioning but compensate at different rates. Better compensation of males contributed to a higher nestling survival success than that of females (Wiebe, 2005). The different limitations of parental feeding capacity in both sexes and the reliance on care from one sex were proposed to be the main causes. However, we found that both male and female Chinese penduline tits showed similar facultative adjustments in compensation for partner removal. Zheng et al. (2021) also reported comparable offspring survival success in male‐only and female‐only cared nests. Therefore, males and females might equally evolve a high ability to solely and successfully provision offspring. The reason why female‐only care nests are more prevalent than male‐only care nests in the population may be caused by other traits that differ between sexes during the entire life history, such as sex bias in mortality rate or adult sex ratio (Fromhage & Jennions, 2016). However, the low recruitment rates of Chinese penduline tits (about 7%–13%, Zheng J unpublished data) did not allow us to conduct investigations on the life‐history trait of sex‐specific mortality, which also impeded the comparison of differences between uniparental care and biparental care nests in trade‐offs between investment and mortality.

### 4.4 Insights from individual variations in compensation after partner removal

Although our results generally found that the remaining parent fully compensated for feeding and barely for brooding after removing one of the parents, the responses between individuals were variable. Individual variations in compensation indicate that the solution towards the abovementioned trade‐off between feeding and brooding largely relies on individual preferences. A study of chestnut thrushes (*Turdus rubrocanus*) showed that the similarity of personality contributes to individual variations in compensation after removing the partner (Lou et al., 2021). Pairs with similar personalities could be better parents, which may result in higher reproductive success (Schuett et al., 2011). In addition, compatible and well‐coordinated pairs may modulate the intensity of sexual conflict within pairs, as the more assortative mates would experience less conflict due to the equal labour divisions during breeding (Baldan & Griggio, 2019; Lou et al., 2021). Parents may also respond differently to mate removal due to their body condition and capability of dealing with stress (Lendvai & Chastel, 2008; Whittingham et al., 1994). However, beyond evaluating the average response at the population level, further experiments are needed to explore the pattern of parental compensation by correlating mate similarity, coordination of partnership and physiological conditions.

## 5 CONCLUSIONS AND FUTURE RECOMMENDATIONS

Our study found that in Chinese penduline tits, parents from uniparental and biparental care nests differ in solving multiple parental care tasks. Parents in biparental nests face a trade‐off while parenting alone, such that they need to put more effort into the task, which more significantly influences offspring fitness, but this restriction is eliminated for parents in uniparental nests. To the best of our knowledge, our study is the first to experimentally reveal the potential role of parental capability in care decisions in species that undergo offspring desertion. Further studies can investigate the consequence of undercompensated brooding duration on nestling fitness. In addition, we suggest that more understanding is needed for the rules of parents allocating effort to multiple parental tasks in response to changed breeding conditions.

## AUTHOR CONTRIBUTIONS

**Jia Zheng:** Conceptualization (lead); data curation (lead); formal analysis (lead); funding acquisition (equal); investigation (equal); methodology (lead); project administration (equal); resources (equal); supervision (equal); writing – original draft (lead); writing – review and editing (equal). **Hui Wang:** Investigation (equal); project administration (equal); writing – review and editing (supporting). **Jiayao Jiang:** Data curation (equal). **Maaike A. Versteegh:** Formal analysis (equal); writing – review and editing (equal). **Zhuoya Zhou:** Investigation (equal); project administration (equal). **Zhengwang Zhang:** Funding acquisition (equal); supervision (equal); writing – review and editing (equal). **De Chen:** Funding acquisition (equal); writing – review and editing (equal). **Jan Komdeur:** Methodology (equal); supervision (lead); writing – review and editing (lead).

## CONFLICT OF INTEREST STATEMENT

The authors declare no competing interests.

## Supporting information


Appendices S1–S4

## Data Availability

Data available from the Dryad Digital Repository https://datadryad.org/stash/share/BkIxkEkI8‐NGZy0rXIjQuhFVtUlP7yf6LyUNTlsbBz0.
