# Supplementary material for: May brood desertion be ruled by partner parenting capability in a polygamous songbird? An experimental study
Source: Ecol Evol. 2024 May 13;14(5):e11394. doi: 10.1002/ece3.11394 (PMC11090777; doi:10.1002/ece3.11394)

**Supplementary materials**

**S1**. The procedure of model selections. The predictors that may affect the response variable were considered in the full models. The models at the first row with the smallest AICc (ΔAICc < 2) and these included the predictors we were most interested in were selected as the final model.

| **Response variable** | **Explanatory variables** | **df** | **Log Likelihood** | **AICc** | **ΔAICc** |
| --- | --- | --- | --- | --- | --- |
| Feeding frequency | Care type + Year + Nestling age | 6 | -96.8 | 197.4 | 0 |
|  | Care type + Number of nestlings + Year + Nestling age | 7 | -97.2 | 197.6 | 0.2 |
|  | Care type + Number of nestlings + Year + Nestling age + Care type × Nestling age | 8 | -99.27 | 200 | 2.6 |
| Feeding frequency | Sex + Nestling age | 5 | 69.72 | 138.8 | 0 |
|  | Sex + Nestling age + Sex × Nestling age | 6 | -71.51 | 139.6 | 0.8 |
|  | Sex + Nestling age + Year + Number of nestlings | 7 | -71.7 | 142.0 | 3.2 |
|  | Sex + Nestling age + Year + Number of nestlings + Sex × Nestling age | 4 | -73.5 | 143.0 | 4.2 |
| Log(brooding duration) | Care type + Nestling age | 5 | -75.2 | 165.3 | 0 |
|  | Number of nestlings + Nestling age | 5 | -77.4 | 165.7 | 0.4 |
|  | Care type + Number of nestlings + Nestling age + Number of nestlings × Nestling age | 7 | -75.7 | 167.1 | 1.8 |
|  | Care type + Number of nestlings + Nestling age | 6 | -77.2 | 167.6 | 2.3 |
|  | Care type + Number of nestlings + Nestling age + Year | 7 | -75.7 | 169.6 | 3.7 |
| Log(brooding duration) | Nestling age + Sex | 5 | -105.0 | 221.0 | 0 |
|  | Sex + Number of nestlings + Nestling age | 6 | -104.7 | 222.7 | 1.7 |
|  | Sex + Number of nestlings + Nestling age + Year | 7 | -104.7 | 225.2 | 4.2 |
| Provisioning frequency | Manipulation stage | 4 | -19.9 | 46.7 | 0 |
|  | Manipulation stage + Subject | 5 | -19.8 | 49.8 | 3.1 |
|  | Manipulation stage + Subject + Manipulation × Subject | 5 | -19.8 | 49.8 | 3.1 |
|  | Manipulation stage + Subject + Manipulation × Subject + Number of nestlings | 6 | -20.4 | 51.8 | 5.1 |
| Log (brood duration) | Manipulation stage | 4 | -21.88 | 54.1 | 0 |
|  | Manipulation stage + Subject | 5 | -20.95 | 55.7 | 1.6 |
|  | Manipulation stage + Subject + Manipulation × Subject | 6 | -20.0 | 57.7 | 3.6 |
|  | Manipulation stage + Subject + Manipulation × Subject + Number of nestlings | 7 | -19.2 | 60.4 | 6.3 |

**S2.**  Variations of individual provisioning response before and after parental removal of biparental nests in Chinese penduline tits. (a) Total feeding frequency (male + female = blue dots) before the experiment and feeding frequency by the remaining parent (red dots = remaining females; green dots = remaining males); (b) the feeding frequency of the remaining parent before and after partner removal (red dots = female feeding frequency before and after the male was removed; green dots = male feeding frequency before and after the female was removed. Each dot indicates the feeding frequency from one nest).

(b)

(a)


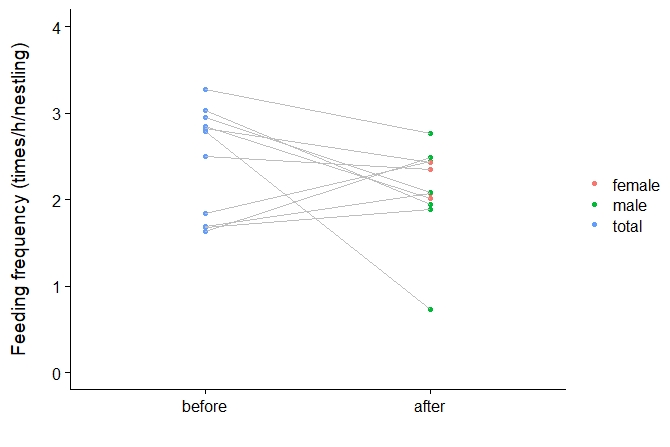

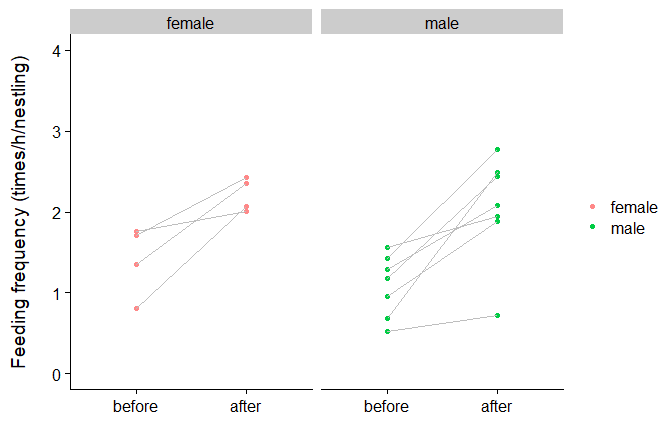


**S3.** Variations of individual brooding response before and after parental removal of biparental nests in Chinese penduline tits. (a) Total brooding duration (male + female = blue dots) before the experiment and brooding duration by the remaining parent (red dots = remaining females; green dots = remaining males); (b) the brooding duration of the remaining parent before and after partner removal (red dots = female brooding duration before and after the male was removed; green dots = male brooding duration before and after the female was removed. Each dot indicates the brooding duration from one nest).


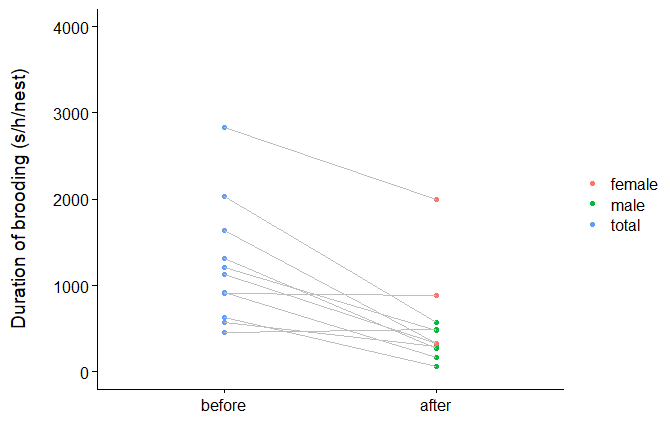

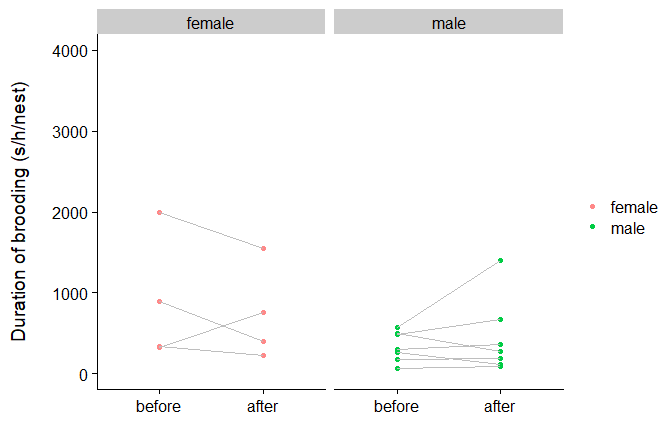


(a)

(b)

**S4.** Testing of statistical robustness using Bayesian modeling analysis. Statistical models in the main contents were simulated in the same modelling structures (from Table 1 to Table 6) by using Bayesian linear multilevel models models. The estimation and posterior distributions were presented as the result for each model.

The list of priors:

| Variables | Priors |
| --- | --- |
| Care type | Normal~ (0,1) |
| Day age | Normal~ (0,3) |
| Year | Normal~ (0,1) |
| Manipulation stage | Normal~ (0,1) |
| Log(Broodduration) | Gamma~(1,0.1) |
| Feeding frequency | Normal~ (2.5,1) |

**Model 1: Feeding frequency~ Care type+Year+Dayage+(1|Nest ID)**

**Table 1**

|  | Estimate | Est.Error | I-95% CI | u-95% CI | Rhat | Bulk_ESS | Tail_ESS |
| --- | --- | --- | --- | --- | --- | --- | --- |
| Intercept | 2.49 | 0.36 | 1.79 | 3.20 | 1.00 | 3049 | 1765 |
| Care type | 0.38 | 0.28 | -0.19 | 0.90 | 1.00 | 2343 | 1576 |
| Day age | 0.11 | 0.03 | 0.06 | 0.16 | 1.00 | 5648 | 2616 |
| Year | -0.92 | 0.32 | -1.54 | -0.31 | 1.00 | 1765 | 1122 |


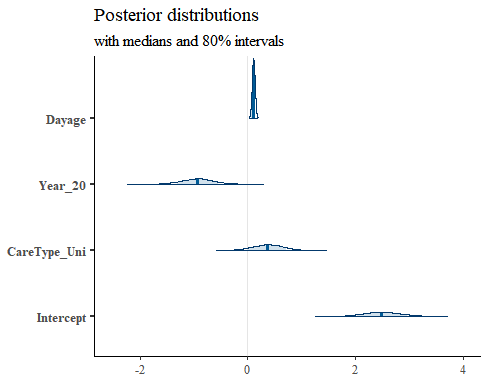


**Model 2: Feeding frequency ~ Sex+ Dayage+ (1|Nest ID)**

**Table 2**

|  | Estimate | Est.Error | I-95% CI | u-95% CI | Rhat | Bulk_ESS | Tail_ESS |
| --- | --- | --- | --- | --- | --- | --- | --- |
| Intercept | 1.00 | 0.20 | 0.62 | 1.38 | 1.00 | 5082 | 2777 |
| Sex | -0.05 | 0.15 | -0.34 | 0.24 | 1.00 | 5395 | 2909 |
| Day age | 0.05 | 0.02 | 0.02 | 0.08 | 1.00 | 5013 | 3068 |


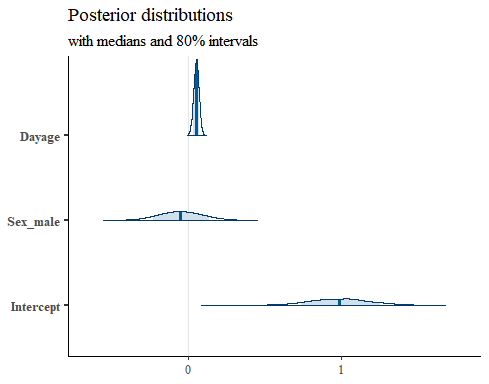


**Model 3: log(Brooding duration) ~ Care type+ Day age+ (1|Nest ID)**

**Table 3**

|  | Estimate | Est.Error | I-95% CI | u-95% CI | Rhat | Bulk_ESS | Tail_ESS |
| --- | --- | --- | --- | --- | --- | --- | --- |
| Intercept | 7.86 | 0.26 | 7.35 | 8.37 | 1.00 | 3308 | 1731 |
| Care type | 0.19 | 0.22 | -0.24 | 0.61 | 1.00 | 2535 | 2455 |
| Day age | -0.15 | 0.02 | -0.19 | -0.11 | 1.00 | 6151 | 2626 |


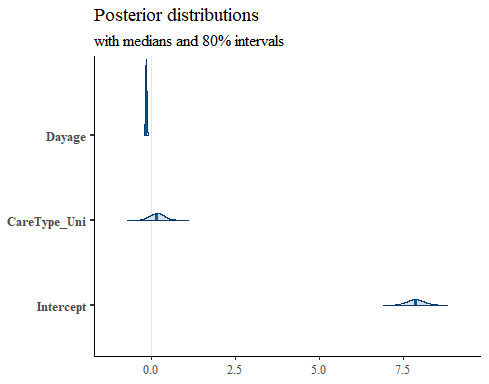


**Model 4: log(Brood duration) ~ Sex+ Dayage +(1|Nest ID)**

**Table 4**

|  | Estimate | Est.Error | I-95% CI | u-95% CI | Rhat | Bulk_ESS | Tail_ESS |
| --- | --- | --- | --- | --- | --- | --- | --- |
| Intercept | 7.61 | 0.33 | 6.96 | 8.25 | 1.00 | 4411 | 1990 |
| Sex | -0.06 | 0.23 | -0.49 | 0.38 | 1.00 | 5214 | 2845 |
| Day age | -0.19 | 0.03 | -0.24 | -0.14 | 1.00 | 6159 | 2999 |


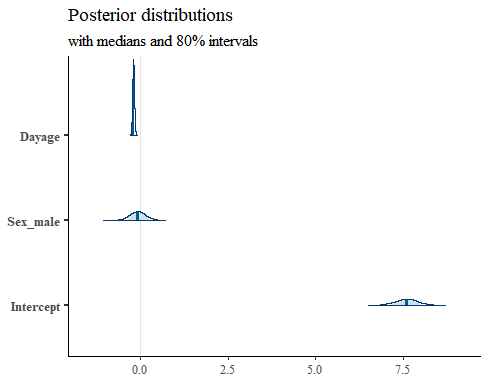


**Model 5: BodyMass~ log(Brooding duration)+ Feeding frequency+ Day age+ (1|Nest ID)**

**Table 5(1).**

|  | Estimate | Est.Error | I-95% CI | u-95% CI | Rhat | Bulk_ESS | Tail_ESS |
| --- | --- | --- | --- | --- | --- | --- | --- |
| Intercept | 3.01 | 0.96 | 1.12 | 4.87 | 1.00 | 2284 | 2699 |
| Log(Brooding duration) | -0.25 | 0.11 | -0.46 | -0.04 | 1.00 | 2513 | 2895 |
| Feeding frequency | 0.58 | 0.08 | 0.43 | 0.74 | 1.00 | 3171 | 2879 |
| Day age | 0.36 | 0.02 | 0.31 | 0.40 | 1.00 | 2647 | 2773 |

**
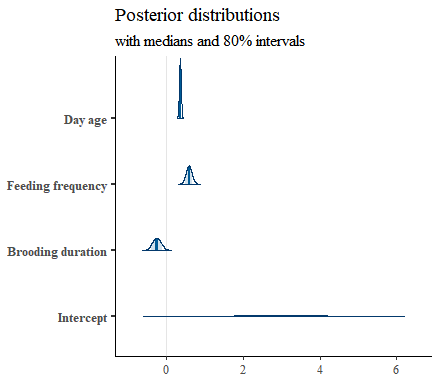
**

**Model 6: [FledgeN|trials(HatchN)]~ log(Brooding duration)+ Feeding frequency+ (1|Nest ID)**

**Table 5(2)**

|  | Estimate | Est.Error | I-95% CI | u-95% CI | Rhat | Bulk_ESS | Tail_ESS |
| --- | --- | --- | --- | --- | --- | --- | --- |
| Intercept | 3.32 | 1.15 | 1.27 | 5.77 | 1.00 | 1078 | 1530 |
| Log(Brooding duration) | 0.05 | 0.83 | -1.55 | 1.68 | 1.00 | 3856 | 3020 |
| Feeding frequency | 0.02 | 0.18 | -0.34 | 0.38 | 1.00 | 3957 | 3217 |


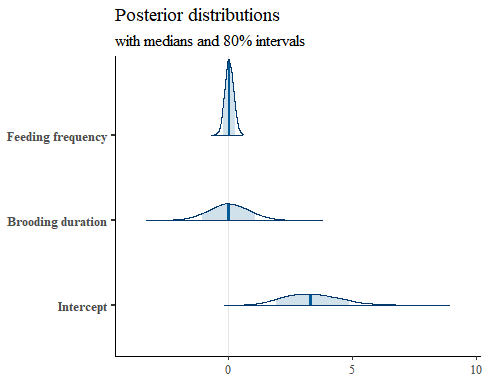


**Model 7: Feeding frequency ~ Manipulation stage + Sex + (1|Nest ID)**

**Table 6(1)**

|  | Estimate | Est.Error | I-95% CI | u-95% CI | Rhat | Bulk_ESS | Tail_ESS |
| --- | --- | --- | --- | --- | --- | --- | --- |
| Intercept | 2.43 | 0.24 | 1.96 | 2.89 | 1.00 | 3024 | 2201 |
| Manipulation stage | -0.35 | 0.26 | -0.85 | 0.17 | 1.00 | 4238 | 2575 |
| Sex | 0.09 | 0.35 | -0.58 | 0.77 | 1.00 | 2384 | 1880 |


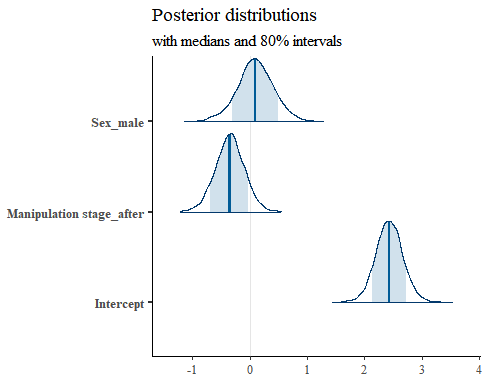


**Model 8: log(Brooding duration) ~ Manipulation stage + Sex +(1|Nest ID)**

**Table 6(2)**

|  | Estimate | Est.Error | I-95% CI | u-95% CI | Rhat | Bulk_ESS | Tail_ESS |
| --- | --- | --- | --- | --- | --- | --- | --- |
| Intercept | 0.15 | 0.01 | 0.14 | 0.16 | 1.00 | 2697 | 2329 |
| Sex | 0.02 | 0.01 | 0.01 | 0.04 | 1.00 | 4659 | 2797 |
| Day age | 0.02 | 0.01 | -0.04 | 0.00 | 1.00 | 1738 | 1682 |


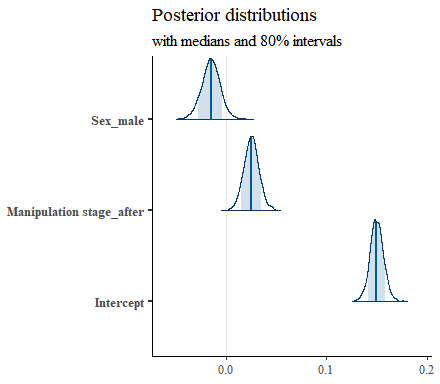

Supplement: Supplementary file 1 — Appendices S1–S4 [file ECE3-14-e11394-s001.docx]
